# Dental Record Books

**Published:** 1857-03

**Authors:** 


### Editorial.

### DENTAL RECORD BOOKS.

We sometime ago received a Dental Record Book of Dr. J. B. Rich, of New York. There is a diagram of the Superior and Inferior circles of teeth upon every alternate page. The teeth are so arranged that every operation can be marked upon them, and its nature indicated by figures and letters. There are spaces on the diagram for the name, age, temperament, and class of teeth. The opposite page is for remarks, descriptions, &c. The arrangement is an excellent one—it presents at a glance all that has been done, and its exact location.

Probably a larger amount and greater variety of operations can be indicated within less compass than by any other method we have seen. We have also received a Case Book, from Dr. H. A. McDaniels, of Nashville Tenn. The arrangement is altogether different from that of Dr. Rich. It is without diagrams, but a very full reference is made to every point of each tooth in the mouth. This is arranged in columns with blank spaces opposite each reference. This is a very comprehensive method.

The second part of the work is a general tablet, upon which, general, transient, and unimportant operations can be noted. The pages for general remarks are in the back part of the book. This work is gotten up in neat style. It can be obtained of the author, or at the Dental depots in this city.

We have noticed a third form of Case Book, in which the labial and buccal portions only of the teeth are exhibited. In this work there are columns for keeping all accounts with the patients. By using this the operator is relieved from keeping any other account books. There are, however, no pages for extended remarks or descriptions. This work is for sale, in two or three styles of binding, by J. T. Toland, of this city.

Of the importance of keeping some systematic record of all operations performed, no one can doubt for a moment. Some look upon it as valuable only so far as it enables the operator to recognize his own operations, that he may not be imposed upon. Superior operators can almost always recognise their own work at a glance. But there is a far more important consideration than simply to avoid imposition—that we may observe the permanency and efficiency of our work.

It is important to indicate how each operation is performed, and then note its efficiency. This should be done in all common operations, and if there is a failure, ascertain the true cause—whether it is from the imperfect manner in which the work was done, or whether it is from an accumulation of injurious agents arising in the mouth subsequent to the operation; or whether there is a strong predisposition in the teeth to decay.

But especially should cases be definitely recorded for observation of new or untried modes of practice. It is only by this means that we can test the merit of untried methods.

If all would pursue a course of this kind, keeping a perfect record of all operations, and observing from time to time, and noting the observations, there would be an accumulation of facts that would serve as a firm basis for correct dental practice. T.

